# Effects of a people care-based song-centered group music program on perceived parental care, self-esteem, peer relationships, and happiness in school-age children

**DOI:** 10.3389/fpubh.2026.1972317

**Published:** 2026-09-29

**Authors:** Su-Hee Kim

**Affiliations:** Department of Nursing, Baekseok University, Cheonan, Republic of Korea

**Keywords:** child, community health services, empathy, happiness, interpersonal relations, music therapy, nursing care, self-concept

## Abstract

**Introduction:**

In the Republic of Korea, the expansion of dual-income households and the nuclearization of families have widened gaps in child care, increasing the role of community care providers; however, how the care they provide is actually perceived by children has rarely been examined. This study developed a People Care-Based Song-Centered Group Music Program that integrates the 10 techniques of interpersonal caring theory into song-centered music activities, and examined its effects on perceived parental care, self-esteem, peer relationships, and happiness in school-age children using a single-group pre-post design. We further explored, as subgroup analyses, the extent to which care was delivered by technique, how outcomes responded at the individual level, and the degree of agreement between children and care providers.

**Methods:**

Thirty school-age children attending community child centers in Daejeon and Chungcheongnam-do were enrolled. A program developed following the analysis, design, development, implementation, and evaluation model was delivered in 10 50-min sessions twice weekly. Data were collected 1 week before the program, immediately after, and 2 weeks after completion via self-report questionnaires and analyzed with one-way repeated-measures analysis of variance.

**Results:**

All five outcomes improved significantly across time (all *p* < 0.001, partial eta-squared 0.80–0.96), and the effects were maintained until 2 weeks after completion. Subgroup analyses revealed three patterns: care delivery differed by technique (praising, forgiving, and accepting rose most, whereas noticing rose least); responder rates differed markedly by outcome (happiness 100% versus self-esteem 40%); and, immediately after the program, care providers rated care significantly higher than children did (difference 0.42 points, *p* = 0.003), particularly for emotionally oriented techniques.

**Conclusion:**

This program may serve as an intervention to enhance the emotional well-being of school-age children in community care settings, and the subgroup findings offer a new perspective on training priorities for care personnel and on the perception gap in emotional care.

## 1 Introduction

The Republic of Korea has entered a super-aged society with one of the lowest fertility rates in the world. The total fertility rate declined from 0.78 in 2022 to 0.72 in 2023, and the total population has been shrinking since it peaked in 2020 (1, 2). At the same time, the growing participation of women in the labor force, the increase in dual-income households, and the nuclearization of families have rapidly changed the child-rearing environment and widened gaps in care (3, 4). In response, the government has expanded the Neulbom School program nationwide, integrating after-school classes and care services; however, the definition of care and the role of care providers under this system remain unclear, and confusion persists in the field (5–7).

Care is defined as the essence of professional nursing, an act accompanied by empathy and emotional communication rooted in love for human beings (8). For children, how they perceive the care they receive from their parents, that is, perceived parental care, plays an important role; children who perceive that they are sufficiently cared for adapt better to physical and emotional difficulties (9, 10). School age is a period in which self-esteem, peer relationships, and happiness are formed together, yet the life satisfaction of Korean children in 2023 remained low among the member countries of the Organisation for Economic Co-operation and Development (11–14). In this context, interpersonal caring, a compassion-based therapeutic practice developed in the Republic of Korea, comprises 10 techniques: noticing, participating, sharing, active listening, being a companion, praising, comforting, instilling hope, forgiving, and accepting (10). This approach has shown positive effects on the self-esteem, interpersonal relationships, and emotional intelligence of individuals with mental illness and of the general population (10, 15–19), and, in children, drum and Orff music programs have improved self-esteem and happiness (18, 19).

The four outcomes addressed in this study are core developmental attributes of school age. Happiness is a key determinant of well-being; for school-age children it positively shapes adolescence and later life, yet the life satisfaction of Korean children, although gradually rising, remains the lowest among children of the member countries of the Organisation for Economic Co-operation and Development, with academic stress, insufficient family communication, and emotional instability from excessive parental expectations cited as contributing factors (11–14). Self-esteem, the individual’s perception of their own worth, enables children to cope with and resolve challenges, and is higher among those with stable attachment to parents and peers. Peer relationships are especially important during school age, often called the peer period, when a sense of belonging within a group is formed; stronger parental attachment and involvement are associated with better peer relationships, and children who fail to acquire diligence and a sense of achievement may develop feelings of inferiority that hinder connection with peers. Perceived parental care, the child’s own perception of the care received, underlies all of these attributes, as children who feel sufficiently cared for adapt better to physical and emotional difficulties (9, 10).

However, three gaps exist in previous research. First, interventions applying the 10 techniques of interpersonal caring to school-age children are limited. Second, previous studies treated the 10 techniques as a single bundle and reported effects only as total scores, providing no information on which care is actually delivered and to what extent, or on how responses differ by outcome. Third, although care is a reciprocal phenomenon established between provider and recipient, few studies have examined the degree of agreement between the two parties using paired data.

Accordingly, this study developed a People Care-Based Song-Centered Group Music Program and verified its effects. The specific aims were, first, to examine the effects of the program on perceived parental care, self-esteem, peer relationships, and happiness in school-age children across time, and second, to explore, through subgroup analyses, the extent of care delivery by technique, the pattern of individual-level responses, and the degree of agreement between children and care providers, thereby elucidating how the program works.

## 2 Materials and methods

### 2.1 Study design

This was a single-group pre-post intervention study designed to examine the effects of a People Care-Based Song-Centered Group Music Program. Without a separate control group, outcome variables were repeatedly measured at three time points (pre, immediately post, and 2 weeks post) in a single group that participated in the program. Reporting followed the Transparent Reporting of Evaluations with Nonrandomized Designs (TREND) guideline.

### 2.2 Participants and sample size

Participants were elementary school children in grades 2 to 6 enrolled at community child centers in Daejeon and Chungcheongnam-do. Ages are reported here as Korean age (a counting system in which a child is 1 year old at birth); the corresponding international ages ranged from approximately 8 to 12 years, and two children in grade 2 were included accordingly. Inclusion criteria were children who understood the purpose of the study, submitted written consent from a legal representative, and were able to participate in music activities; children taking specific medications for health reasons were excluded.

The sample size was calculated using G*Power 3.1 (Heinrich Heine University, Dusseldorf, Germany). For a single-group repeated-measures analysis of variance with an effect size f of 0.25, a significance level of 0.05, a power of 0.80, three repeated measurements, and a correlation among measurements of 0.50, a minimum of 28 participants was required. Considering attrition, 30 children were recruited, and all 30 completed the study; the participant flow is shown in Figure 1.

**Figure 1:**
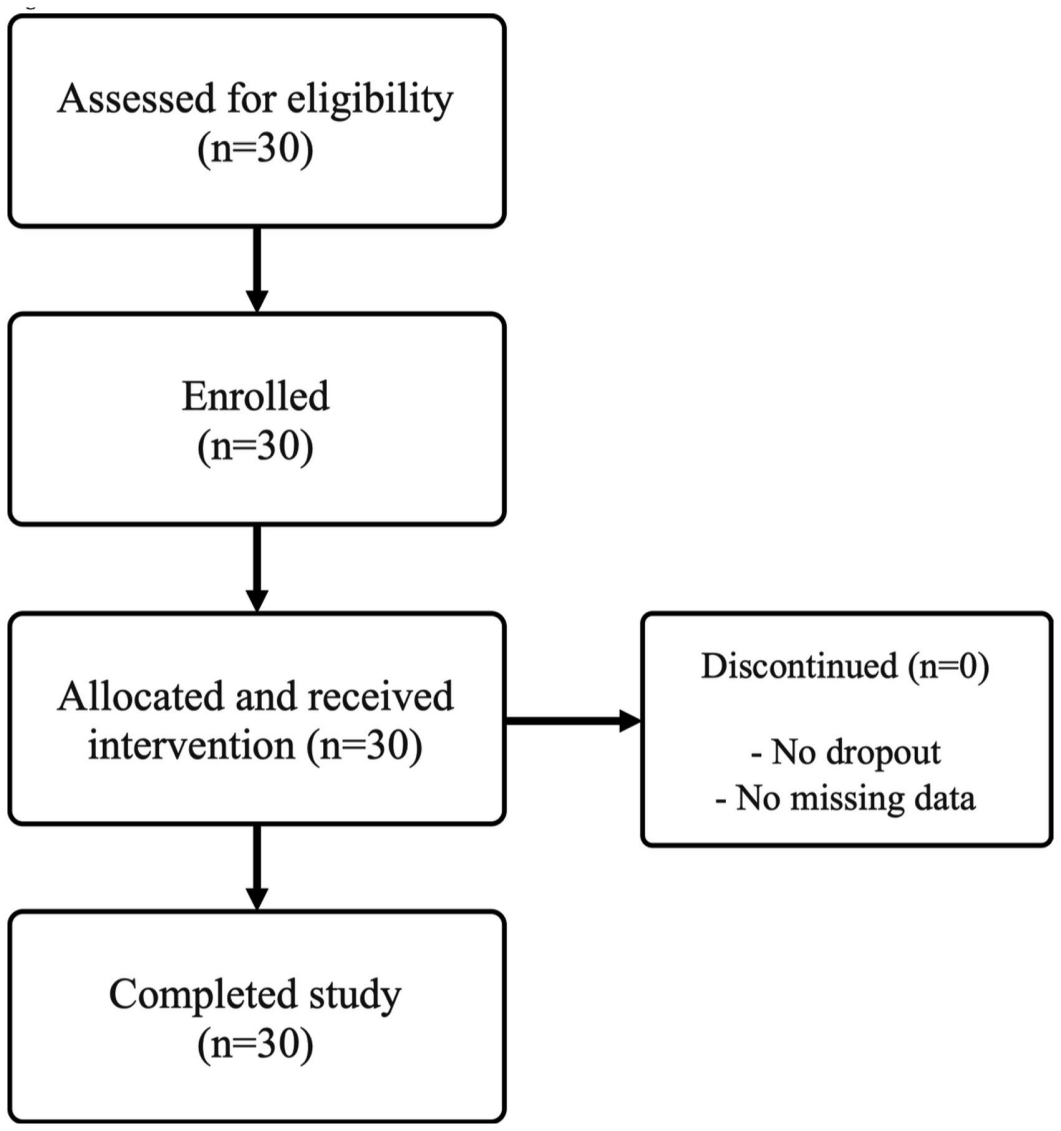
Participant flow. Under the single-group design, 30 children were enrolled and all completed the intervention, with no dropout and no missing data.

### 2.3 Intervention program

This program is a song-centered group music activity designed to improve school-age children’s self-esteem, happiness, peer relationships, and perceived parental care by integrating the 10 techniques of interpersonal caring theory into music activities (9, 10). The program was developed following the analysis, design, development, implementation, and evaluation (ADDIE) instructional design model (20), and its framework was organized into three core dimensions, theme, harmony, and rhythm, based on the metaphorical expression of care through musical elements (21). Three care techniques important for the well-being of school-age children, comforting, noticing, and instilling hope, were assigned to these three dimensions with three sessions each, and a final integrative session was added for a total of 10 sessions. The rhythm dimension in particular emphasizes that caregiving actions between provider and child should occur regularly and continuously like the rhythm of music, and the 10 care techniques were designed to be applied repeatedly in every session.

Each session proceeded in three phases: introduction, development, and conclusion. In the introduction, a greeting song composed by the researcher to reflect the session objectives established motivation to participate and led the children into song activities aligned with the session topic. In the development phase, the 10 care techniques were applied through full-fledged group music activities such as singing, songwriting, appreciation of preferred songs, playing melodic and non-melodic percussion instruments, and ensemble performance. In the conclusion, the session ended with a closing song composed by the researcher and the sharing of reflections, followed by planning for the next session. Groups were limited to 10 children or fewer to ensure sufficient interaction. The specific composition of the program is presented in Table 1.

**Table 1:** Composition of the people care-based song-centered group music program.

Dimension (sessions)	Matched care technique	Main objective
Theme (sessions 1–3)	Comforting	Express care in a warm, positive manner to provide emotional stability and comfort
Harmony (sessions 4–6)	Noticing	Achieve agreement and synchronization through communication between provider and child
Rhythm (sessions 7–9)	Instilling hope	Engender hope through regular and continuous interaction
Integration (session 10)	All 10 techniques	Integrative activities combining the three dimensions and closure

The program was developed following the ADDIE instructional design model and is based on the metaphorical expression of care through musical elements and the 10 techniques of interpersonal caring. Each session comprised three stages (introduction, development, conclusion), with groups of 10 children or fewer, delivered twice weekly for 50 min over 10 sessions.

#### 2.3.1 Program development

The program was developed following the five stages of the ADDIE instructional design model. In the analysis stage, the characteristics and care needs of school-age children were identified through a literature review. In the design stage, session objectives, operation methods, and evaluation tools were selected based on the analysis, and the three-dimensional structure and three-phase session format were established. In the development stage, care techniques and Orff music activities were combined for each dimension: the theme dimension centered on comforting through singing, appreciation, and physical activity; the harmony dimension centered on noticing through ensemble and instrument playing; and the rhythm dimension centered on instilling hope through regular interaction.

The content validity of the developed program was evaluated by five experts: one professor from a department of nursing, one professor of psychiatric nursing, one music therapist, one mental health nurse, and one music therapy instructor with a doctorate and program development experience. Using the content validity index, the program objectives, content, structure, operating time, application methods, and evaluation tools were rated on a four-point scale (22). Based on expert feedback, the technique titles were revised to be more child-friendly and a variety of melodic and non-melodic percussion instruments were incorporated. A pilot study was then conducted in May 2023 with 10 children from a community child center who had no prior experience with music programs. The program was revised and finalized by incorporating the children’s feedback and the observational evaluations of a mental health nurse, a music therapy expert, and three care providers from the center (23–26).

The final program was implemented at four community child centers from June to August 2023. It was delivered in 10 50-min sessions twice weekly, with each group limited to 10 children or fewer. The intervention was delivered by a researcher holding a doctorate in psychiatric nursing and a master’s degree in music therapy, with a trained mental health nurse serving as a co-therapist. All sessions were video-recorded and reviewed. To maintain treatment fidelity across the four centers, all sessions were delivered by the same researcher and co-therapist using a standardized session protocol, and the video recordings were reviewed to confirm consistent delivery across sites.

### 2.4 Measures

Self-esteem was measured with the Rosenberg Self-Esteem Scale (27), peer relationships with the peer relationship scale for children and adolescents (28), and happiness with the 30-item happiness scale for elementary school students (29), which comprises four subdomains: home environment, self-competence, competence, and interpersonal relationships. Perceived parental care was measured with a 10-item parental caring awareness scale for elementary school students, adapted and supplemented (30), in which each item corresponds to one of the 10 care techniques. In this study, the care providers were those who directly cared for the children during the program, namely their parents and the community child center teachers who assume a parental caregiving role in community care settings; the same 10 items were therefore administered to both children and these care providers to obtain paired data on the perceptions of the two parties. For all instruments, higher scores indicate higher levels of the attribute. In the present sample, Cronbach’s alpha at baseline was 0.727 for self-esteem, 0.912 for peer relationships, 0.768 for happiness, 0.764 for child-rated perceived parental care, and 0.693 for provider-rated perceived parental care. For the Rosenberg Self-Esteem Scale, the two negatively worded items were reverse-scored; without reverse-scoring, the alpha was 0.472, and reverse-scoring these two items raised it to 0.727, consistent with the intended scale structure.

Each scale used a Likert format in which higher scores indicate higher levels of the measured attribute. The self-esteem scale assesses the child’s overall sense of self-worth, the peer relationship scale captures the quality of interaction and belonging among peers, and the happiness scale comprises four subdomains: home environment, self-competence, competence, and interpersonal relationships. The perceived parental care scale consists of 10 items, each corresponding to one of the 10 interpersonal caring techniques, so that the degree of delivery of each technique could be examined individually. Administering the identical 10 items to both children and care providers allowed the perceptions of the two parties to be compared directly at the level of each technique.

### 2.5 Data collection and ethical considerations

This study was approved by the Institutional Review Board of Dankook University (DKU 2023–06-035, approved on June 30, 2023). All data collection began after IRB approval: the pretest was administered from July 2023, 1 week before the program, followed by posttests immediately after and 2 weeks after completion, using self-report questionnaires. Written consent was obtained from the legal representatives of the children and written assent from the children. The study was conducted in accordance with the principles of the Declaration of Helsinki.

### 2.6 Data analysis

All statistical analyses were performed with R 4.3.3 (R Foundation for Statistical Computing, Vienna, Austria). Changes across time in the outcome variables were tested with one-way repeated-measures analysis of variance, and effect sizes were reported as partial eta-squared. For significant main effects, *post hoc* comparisons were conducted with paired *t*-tests for pre versus post 1, pre versus post 2, and post 1 versus post 2. The Rosenberg Self-Esteem Scale was scored with reverse-coded items handled accordingly.

The following three subgroup analyses were conducted. First, the pre to post 1 increase for each of the 10 care techniques was calculated to compare the extent of care delivery by technique. Second, the proportion of children who improved by at least one point from baseline was defined as the responder rate and calculated for each outcome; on the five-point Likert metric used here, a one-point gain represents a shift of at least one response category and was adopted as a conservative, interpretable threshold for a meaningful individual change. This responder rate is an exploratory descriptive indicator rather than a validated clinical cut-off. Third, the mean difference and correlation between paired child and care-provider ratings were analyzed as the perception gap and agreement. In addition, correlations among the change scores of the outcome variables were computed to describe the co-occurrence of changes. Because the subgroup analyses were exploratory, no correction for multiple comparisons was applied, and the results were interpreted at the level of hypothesis generation. The significance level for all tests was 0.05.

## 3 Results

### 3.1 Participant characteristics

The participants were 30 elementary school children in grades 3 to 6. Their general characteristics are presented in Table 2.

**Table 2:** General characteristics of participants (*n* = 30).

Characteristic	Category	*n* (%)
Grade	Grades 3–4	16 (53.3)
	Grades 5–6	14 (46.7)
Sex	Boys	4 (13.3)
	Girls	26 (86.7)
Family type	Single-parent	4 (13.3)
	Two-parent	26 (86.7)
Father age	40–49 y	15 (50.0)
	≥50 y	15 (50.0)
Mother age	30–39 y	29 (96.7)
	40–49 y	1 (3.3)

Values are frequency (percentage). Two second-grade children were included in the grades 3–4 group.

### 3.2 Effects of the program

One-way repeated-measures analysis of variance showed a significant main effect of time for all five outcome variables (*p* < 0.001) (Table 3 and Figure 2). Self-esteem increased from 2.00 at baseline to 2.88 at post 1 and 2.87 at post 2 [*F*(2, 58) = 115.29, partial eta-squared = 0.799]; peer relationships from 2.22 to 3.54 and 3.54 (*F* = 254.62, partial eta-squared = 0.898); and happiness from 1.47 to 3.54 and 3.58 (*F* = 711.37, partial eta-squared = 0.961). Perceived parental care rated by children increased from 1.71 to 3.52 and 3.76 (*F* = 183.05, partial eta-squared = 0.863), and that rated by care providers from 1.71 to 3.94 and 4.04 (*F* = 527.03, partial eta-squared = 0.948).

**Table 3:** Changes over time, one-way repeated-measures ANOVA, and *post hoc* comparisons (*n* = 30).

Variable	Pre *M* ± SD	Post 1 *M* ± SD	Post 2 *M* ± SD	*F*	eta2*p*	Pre-Post 1 t (dz)	Pre-Post 2 t (dz)	Post 1-Post 2 t (p)
Self-esteem	2.00 ± 0.36	2.88 ± 0.26	2.87 ± 0.27	115.29***	0.799	10.66*** (1.95)	10.83*** (1.98)	−1.44 (0.161)
Peer relationships	2.22 ± 0.40	3.54 ± 0.38	3.54 ± 0.38	254.62***	0.898	15.99*** (2.92)	15.93*** (2.91)	1.00 (0.326)
Happiness	1.47 ± 0.18	3.54 ± 0.29	3.58 ± 0.42	711.37***	0.961	32.98*** (6.02)	26.47*** (4.83)	0.95 (0.351)
Perceived parental care (child)	1.71 ± 0.39	3.52 ± 0.72	3.76 ± 0.66	183.05***	0.863	12.94*** (2.36)	15.53*** (2.83)	3.70 (<0.001)
Perceived parental care (provider)	1.71 ± 0.31	3.94 ± 0.42	4.04 ± 0.43	527.03***	0.948	23.70*** (4.33)	23.11*** (4.22)	3.69 (<0.001)

Values are mean ± standard deviation. Degrees of freedom for *F* are (2, 58); *post hoc* comparisons are paired *t*-tests (df = 29). dz, Cohen’s dz; eta2p, partial eta-squared. ***p < 0.001. Self-esteem was reverse-scored.

**Figure 2:**
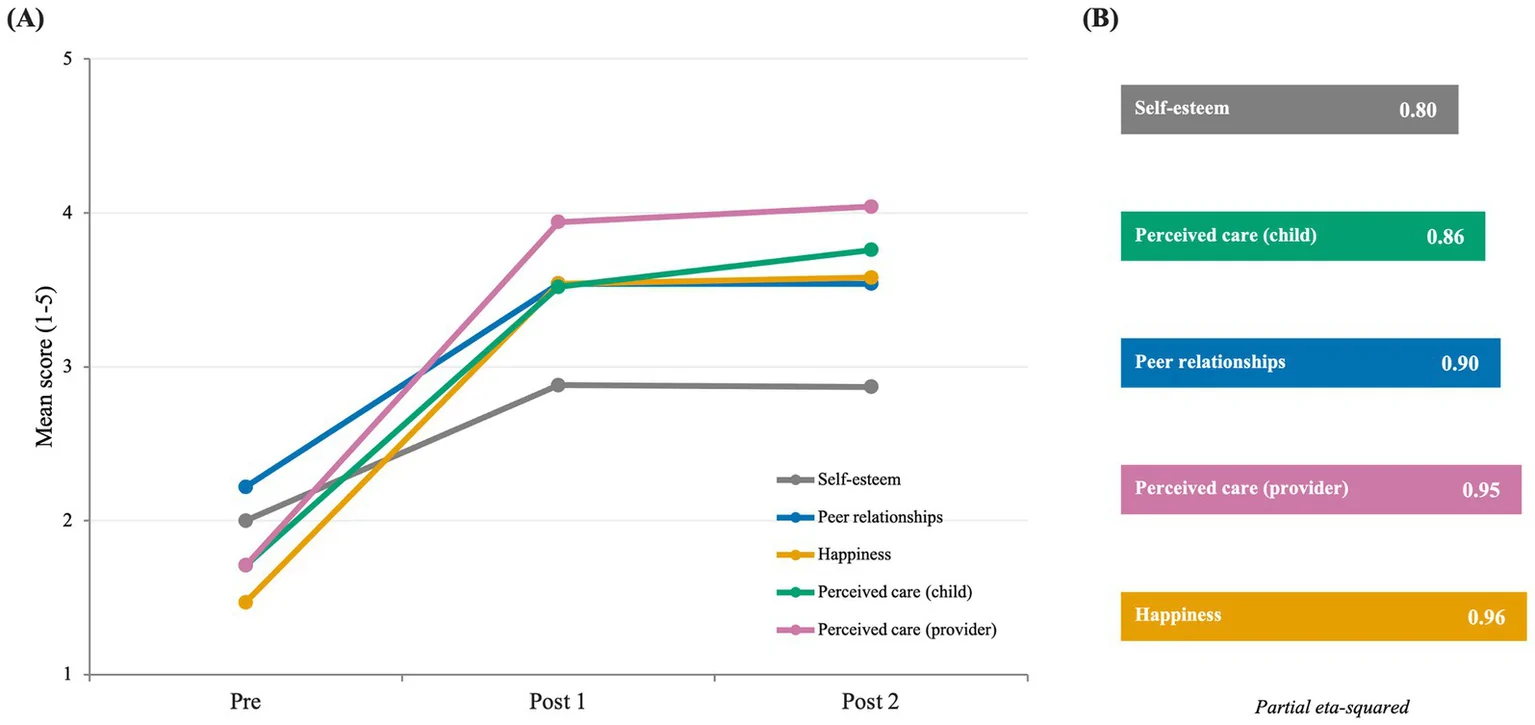
Changes over time in outcome variables **(A)** and effect sizes **(B)**. **(A)** Means and standard deviations of the five outcomes at pre, post 1, and post 2. **(B)** Partial eta-squared for each variable. Self-esteem was reverse-scored.

In *post hoc* comparisons, all five variables were significantly higher at post 1 and post 2 than at baseline (*p* < 0.001). The perceived parental care rated by children and by care providers increased significantly further from post 1 to post 2 (*t* = 3.70, *p* < 0.001; *t* = 3.69, *p* < 0.001), whereas the other three variables did not differ significantly between post 1 and post 2.

### 3.3 Extent of care delivery by technique

The pre to post 1 increase for the 10 care techniques differed by technique (Table 4). The increase was largest for praising (+2.23), forgiving (+2.20), accepting (+2.20), and instilling hope (+2.10), and smallest for noticing (+0.97). Noticing had the highest baseline score among the 10 techniques, at 2.10.

**Table 4:** Extent of care delivery by technique: pre to post 1 change (*n* = 30).

Care technique	Pre *M* ± SD	Post 1 *M* ± SD	Increase	*t*	*p*
Praising	1.40 ± 0.56	3.63 ± 1.03	+2.23	11.76	<0.001
Forgiving	1.33 ± 0.48	3.53 ± 1.01	+2.20	11.69	<0.001
Accepting	1.60 ± 0.56	3.80 ± 1.03	+2.20	11.00	<0.001
Instilling hope	1.60 ± 0.72	3.70 ± 1.06	+2.10	10.22	<0.001
Active listening	1.77 ± 0.63	3.57 ± 1.01	+1.80	7.45	<0.001
Comforting	1.80 ± 0.76	3.53 ± 1.01	+1.73	7.72	<0.001
Participating	1.87 ± 0.78	3.57 ± 1.01	+1.70	6.80	<0.001
Sharing	1.77 ± 0.68	3.40 ± 0.89	+1.63	8.15	<0.001
Being a companion	1.83 ± 0.53	3.40 ± 1.00	+1.57	7.36	<0.001
Noticing	2.10 ± 0.96	3.07 ± 1.17	+0.97	3.43	0.002

Paired *t*-tests, sorted by the size of the increase. Noticing had the highest baseline score and thus the smallest increase.

### 3.4 Individual-level responder analysis

The proportion of children who improved by at least one point from baseline differed by outcome. All children improved on happiness (100%) and on care-provider-rated perceived parental care (100%); 90% improved on child-rated perceived parental care and 73% on peer relationships. On self-esteem, 40% improved by at least one point.

### 3.5 Perception gap between children and care providers

Immediately after the program, perceived parental care was rated 3.52 by children and 3.94 by care providers, with the care-provider rating significantly higher (difference 0.42 points, *t* = 3.27, *p* = 0.003). By technique, significant differences between the two parties were found for comforting (gap +0.67), being a companion (+0.57), praising (+0.57), and instilling hope (+0.53), with the care-provider rating higher in all cases (Table 5); the other six techniques showed no significant difference.

**Table 5:** Perception gap between children and care providers by technique at post 1 (*n* = 30).

Care technique	Child *M* ± SD	Provider *M* ± SD	Gap	*t*	*p*
Comforting	3.53 ± 1.01	4.20 ± 0.61	+0.67	3.01	0.005
Being a companion	3.40 ± 1.00	3.97 ± 0.72	+0.57	2.54	0.017
Praising	3.63 ± 1.03	4.20 ± 0.71	+0.57	2.54	0.017
Instilling hope	3.70 ± 1.06	4.23 ± 0.68	+0.53	2.50	0.018
Forgiving	3.53 ± 1.01	3.97 ± 1.10	+0.43	1.69	0.102
Noticing	3.07 ± 1.17	3.40 ± 0.81	+0.33	1.33	0.194
Participating	3.57 ± 1.01	3.87 ± 0.73	+0.30	1.30	0.204
Active listening	3.57 ± 1.01	3.87 ± 0.94	+0.30	1.22	0.231
Accepting	3.80 ± 1.03	4.07 ± 0.64	+0.27	1.22	0.234
Sharing	3.40 ± 0.89	3.60 ± 0.93	+0.20	1.06	0.297
Total	3.52	3.94	+0.42	3.27	0.003

The gap is the care-provider rating minus the child rating. Paired *t*-tests, sorted by the size of the gap.

### 3.6 Co-occurrence of changes in outcomes

In the correlation analysis of change scores, the change in care-provider-rated perceived parental care was significantly correlated with the change in happiness (*r* = 0.58, *p* = 0.001) (Figure 3), and the changes in child-rated and care-provider-rated perceived parental care were also correlated (*r* = 0.37, *p* = 0.042). The change in self-esteem was not significantly correlated with the change in any other outcome (*p* > 0.05) (Table 6).

**Figure 3:**
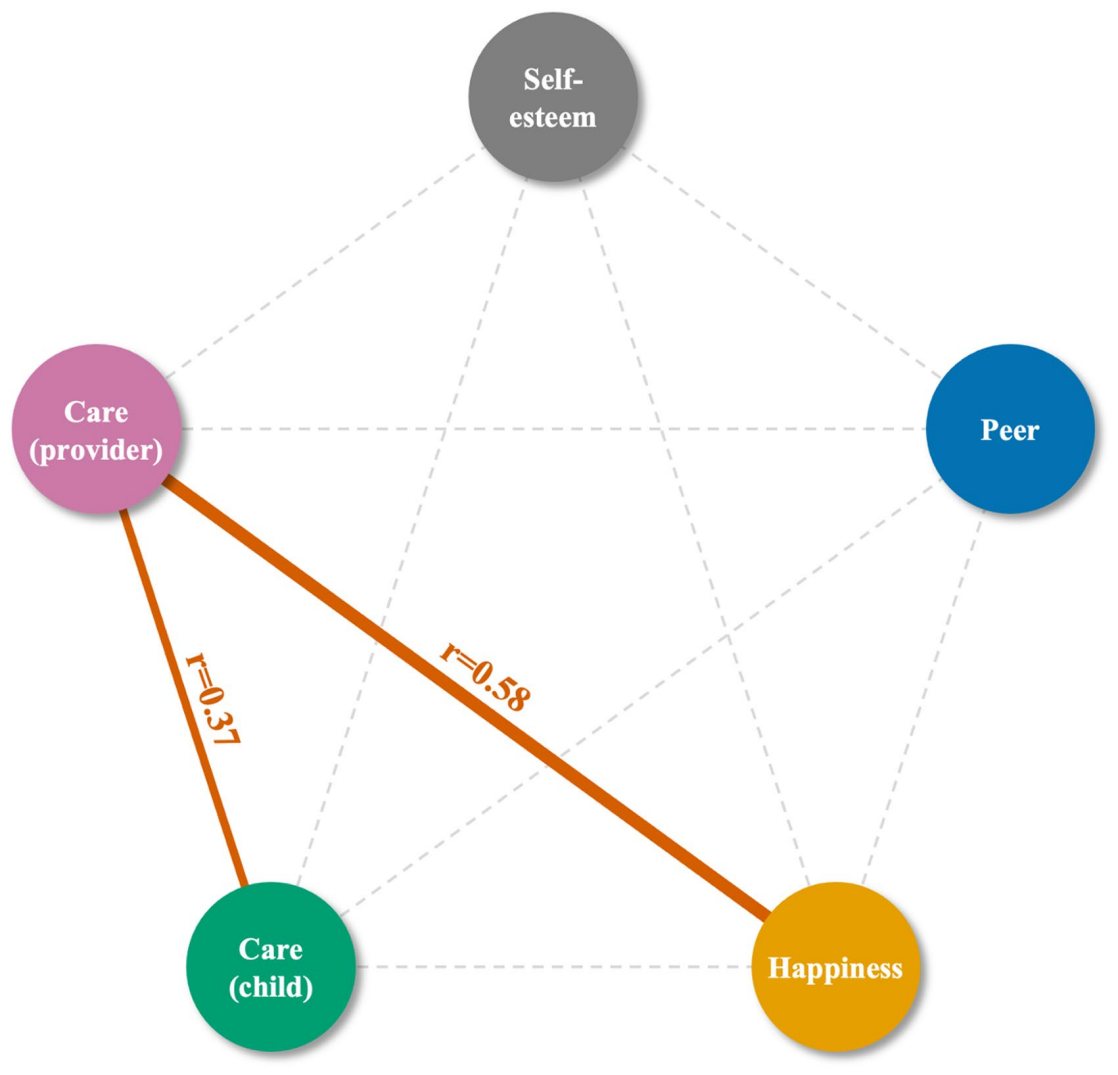
Correlation network of changes in outcomes. Pearson correlations among change scores. Solid lines denote significant correlations (*p* < 0.05) and dashed lines non-significant ones; line thickness indicates the magnitude of the correlation. Correlations describe co-occurrence of changes, not causation.

**Table 6:** Correlation matrix of change scores among outcome variables (*n* = 30).

Variables	Self-esteem	Peer	Happiness	Care (child)	Care (provider)
Self-esteem	–				
Peer	0.30	–			
Happiness	0.11	0.26	–		
Care (child)	0.04	0.26	0.30	–	
Care (provider)	0.14	0.16	0.58*	0.37*	–

Pearson correlations among pre to post 1 change scores. *p < 0.05. Correlations describe co-occurrence of changes, not causation. Self-esteem was reverse-scored.

## 4 Discussion

This study was conducted to examine the effects of a People Care-Based Song-Centered Group Music Program on perceived parental care, self-esteem, peer relationships, and happiness in school-age children attending community child centers, using a single-group pre-post design. The main findings were as follows. All five outcome variables showed a significant main effect of time, with the largest effect sizes for happiness (partial eta-squared = 0.961) and care-provider-rated perceived parental care (partial eta-squared = 0.948) and the smallest for self-esteem (partial eta-squared = 0.799). Subgroup analyses showed that care delivery differed by technique (praising, forgiving, and accepting rose most, and noticing least), that responder rates differed markedly by outcome (happiness 100%, self-esteem 40%), and that care providers rated care significantly higher than children immediately after the program (difference 0.42 points).

The improvements in perceived care, happiness, and peer relationships are consistent with previous studies reporting the effects of interpersonal caring and group music interventions. In previous research, interventions applying interpersonal care improved the interpersonal relationships and care perception of nursing students, elementary school students, and nurses (9, 17, 31–35), and group music activities have been reported to elicit emotional support and empathy through narrative, melody, and ensemble performance (36). Interpreting the present results in light of this literature, children in this program expressed their emotions in a safe form by writing lyrics and completing songs themselves, and the frequency and density of interaction increased as care providers participated in the same process. The regularity and continuity of care emphasized in the rhythm dimension allowed the 10 care behaviors to be experienced repeatedly in every session, which may underlie the further increase in perceived care after the program ended.

The subgroup analyses of this study reveal three points that previous research has not addressed. In previous studies, the 10 techniques of interpersonal caring have been treated as a single bundle and reported only at the total-score level (10, 18, 19), and no study has compared the perceptions of care providers and recipients using paired data. Considering these gaps, the present results can be interpreted as follows. First, the hierarchy of care delivery shows which care the program newly filled; that noticing rose little because it was already high at baseline, whereas praising, forgiving, and accepting rose substantially, suggests that children in community care settings may have received relatively less recognition and acceptance. Second, that only care perception increased further from immediately post to 2 weeks post, whereas the other variables were maintained, suggests that care as a proximal outcome is internalized more deeply over time. Third, that self-esteem had few responders (40%) suggests that self-esteem is more strongly determined by the accumulated interaction and attachment quality with caregivers than by a short-term group experience (37, 38). Fourth, the perception gap in emotionally oriented care (comforting, being a companion, praising, instilling hope; mean +0.58) tended to be larger than that in instrumentally oriented care (+0.31; *p* = 0.086), indicating that the emotional care that providers believe they deliver may not be perceived to the same extent by children.

Integrating these results, the effects of this program appear to follow different response patterns depending on the outcome rather than a single uniform pathway. Happiness and care perception changed immediately and substantially in almost all children, and their change scores were correlated (*r* = 0.58 between care-provider-rated care and happiness); this co-occurrence is descriptive and does not establish a causal or directional link, but it is consistent with the possibility that the delivery of care and emotional well-being tend to move together. In contrast, self-esteem had few responders and was independent of the changes in other outcomes, forming a separate axis that is difficult to reach with a short-term intervention. Practically, this structure suggests priorities for training community care personnel: to enhance emotional well-being and care perception, it is efficient to focus on recognition-type care such as praising, forgiving, and accepting, and to reduce the perception gap in emotional care, a feedback procedure is needed by which providers confirm how their care is perceived by children.

These findings carry several implications for community child care and nursing practice. First, because emotional well-being and care perception responded most strongly, programs for children in community care settings may prioritize recognition-oriented care techniques such as praising, forgiving, and accepting, which showed the largest gains. Second, the perception gap in emotionally oriented care indicates a need for structured feedback in which care providers verify how their care is actually perceived by children, rather than assuming that delivered care is received as intended. Third, the limited responder rate for self-esteem suggests that attributes shaped by cumulative attachment may require sustained rather than short-term intervention, and that music-based interpersonal care could be integrated into ongoing community programs rather than delivered as a one-time course.

The strengths of this study are that it obtained paired data from children and care providers to measure care from both provider and recipient perspectives, analyzed the 10 care techniques at the individual level rather than as a total score, and conducted an individual-level responder analysis beyond the group mean. Several limitations must be emphasized when interpreting these findings. Most importantly, the single-group pre-post design without a control group means that the observed improvements cannot be attributed to the program with confidence, as they may partly reflect maturation, repeated testing, regression to the mean, or Hawthorne effects rather than the intervention itself. We therefore refrain from any causal claim. Although the effects were large (partial eta-squared 0.80–0.96) and differed selectively across outcomes, and although care delivery differed hierarchically by technique, these patterns are only suggestive and cannot by themselves rule out the confounders above. Further limitations include a small sample of 30 children drawn from two regions and a pronounced sex imbalance (86.7% girls), both of which restrict the generalizability of the findings; the reliance on self-report without participant blinding; a follow-up limited to 2 weeks; the modest internal consistency of the self-esteem and provider-rated care scales; and exploratory subgroup analyses conducted without correction for multiple comparisons, whose results should be regarded as hypothesis-generating. Future research should confirm the response structure and perception gap suggested here through (i) designs including a randomized or waitlist control group, (ii) long-term follow-up of 6 months or more, (iii) the use of measures beyond self-report including observer ratings, (iv) tests of mediation and moderation with larger samples, and (v) replication in multicenter samples including vulnerable children.

## 5 Conclusion

The People Care-Based Song-Centered Group Music Program significantly improved perceived parental care, self-esteem, peer relationships, and happiness in school-age children, and the effects were maintained until 2 weeks after completion. Subgroup analyses showed that care delivery differed by technique, that individual-level responses differed markedly by outcome, and that a perception gap in emotional care existed between children and care providers. This program may serve as a nursing intervention to enhance the emotional well-being of school-age children in community care settings, and the subgroup findings offer a new perspective on training priorities for care personnel and on the perception gap in emotional care. Given the limitations of the single-group design, causal interpretation requires caution, and confirmation through subsequent controlled studies is needed.

## Data Availability

The raw data supporting the conclusions of this article will be made available by the authors, without undue reservation.
